# A simple mathematical treatment for predicting linear solvent strength behavior in gradient elution: Application to biomolecules

**DOI:** 10.1002/jssc.202200161

**Published:** 2022-05-26

**Authors:** Davy Guillarme, Thomas Bouvarel, Florent Rouvière, Sabine Heinisch

**Affiliations:** ^1^ School of Pharmaceutical Sciences University of Geneva CMU ‐ Rue Michel Servet 1, 1211 Geneva 4 Switzerland; ^2^ Institute of Pharmaceutical Sciences of Western Switzerland University of Geneva CMU ‐ Rue Michel Servet 1, 1211 Geneva 4 Switzerland; ^3^ Université de Lyon Institut des Sciences Analytiques, UMR 5280, CNRS 5 rue de la Doua Villeurbanne 69100 France

**Keywords:** gradient elution, linear solvent strength parameters, monoclonal antibodies, proteins analysis, retention modeling

## Abstract

This paper describes an approach to rapidly and easily calculate the linear solvent strength parameters, namely log *k*_0_ and S, under reversed‐phase liquid chromatography conditions. This approach, which requires two preliminary gradient experiments to determine the retention parameters, was applied to various representative compounds including small molecules, peptides, and proteins. The retention time prediction errors were compared to the ones obtained with a commercial HPLC modeling software, and a good correlation was found between the values. However, two important constraints have to be accounted for to maintain good predictions with this new approach: i) the retention factor at the initial composition of the preliminary gradient series have to be large enough (i.e., log *k*_i_ above 2.1) and ii) the retention models have to be sufficiently linear. While these two conditions are not always met with small molecules or even peptides, the situation is different with large biomolecules. This is why our simple calculation method should be preferentially applied to calculate the linear solvent strength parameters of protein samples.

**Article Related Abbreviations**
FA: formic acid
HSA: human serum albumin
LSS: linear solvent strength
TFA: trifluoroacetic acid

## 1 INTRODUCTION

The development of retention models in HPLC is crucial for method development strategy. Since the 70s, many attempts have been made to develop retention models. Such models have been included in HPLC modeling software (i.e., Drylab, Chromsword, ACD/LC simulator, Osiris…) [1, 2]. Among the suggested retention models, those based on a linear solvent strength (LSS) behavior are certainly the most frequently applied ones. LSS gradients, originally developed by Snyder and Dolan in the 80s [3] are obtained when the composition of the stronger solvent is a linear function of the time, and the isocratic retention of the solute (log *k*) is a linear function of the volume fraction of the stronger solvent, according to:

(1) $$\log k=\log k_{0}-S\times C$$

where *k* is the retention factor and C, the volume fraction of organic solvent in the mobile phase comprised between 0 and 1. *k*_0_ is the (extrapolated) value of *k* in pure water (C = 0) and S is the solvent strength parameter, which is constant for a given compound and fixed experimental conditions. Log *k*_0_ and S are characteristic of a particular combination of solute, mobile phase, and stationary phase.

Besides linear models, several other retention models have been suggested, such as the quadratic model, adsorption model, mixed‐mode model, and Neue‐Kuss model [4, 5, 6]. However, as summarized in a recent review paper [7], the adsorption and mixed‐mode models are scarcely used under RPLC conditions. On the other hand, a recent work compared the linear, quadratic, and Neue‐Kuss models for small molecules, peptides, and proteins [8]. Despite its simplicity, it appears that the retention time of intact proteins is better described by the linear Equation (1) (lowest retention time prediction errors).

The model coefficients (log *k*_0_ and S) can be experimentally determined from the analyte retention times of two or more isocratic runs (two or more mobile phase compositions) [7]. However, such a strategy is impractical when the retentions of the analytes are too different. Indeed, some of them could elute in the dead volume, while others are too retained by the column. This is particularly true for protein species, which are known to be suitably retained only within a very narrow composition range [9]. Therefore, gradient elution is preferred, so that all analytes are properly eluted during the run. In this context, two preliminary linear gradients with two different gradient times are usually performed to determine the unknown log *k*_0_ and S values. It requires a complex mathematical treatment involving either an iterative numerical procedure, a non‐linear regression technique, or finding the zero of a function [1]. In this context, the use of HPLC modeling software is highly recommended for accurate/rapid determination.

In the present work, we propose an alternative mathematical approach based on a simple linear regression, which can be easily applied with an Excel sheet. It involves two (or more) gradient experiments with varying gradient times. Besides the need to consider a linear model, this approach can only work properly if the retention factors at the initial composition are sufficiently large. Therefore, we checked the method's applicability to a large variety of compounds including small molecules, peptides, and large proteins. The validity of the linear model was checked using a commercial HPLC modeling software (Osiris, Euradif, France). Finally, a simple Excel tool was developed for rapid and accurate calculation of all LSS parameters (the tool is particularly adapted for proteins, as shown in this work), and is freely available on our website.

## 2 THEORETICAL SECTION

### 2.1 Calculation of log *k*_0_ and *S* values

For a given compound analyzed in RPLC under gradient conditions, assuming a linear retention model (Equation (1)), the retention factor at solute elution (*k*_e_) can be expressed against the volume fraction at elution (*C*_e_) as follows:

(2) $$\log\left(k_{e}\right)=\log\left(k_{0}\right)-S\times C_{e}$$

By rearrangement, Equation (2) can be easily transformed into the following expression:

(3) $$C_{e}=\frac{1}{S}\times \log\left(\frac{k_{0}}{k_{e}}\right)$$

Under LSS gradient conditions, *k*_e_ is given by the following equation [10]:

(4) $$k_{e}=\frac{1}{2.3\times b+\frac{1}{k_{i}}}$$

where k_i_ represents the retention factor under initial gradient conditions and *b* is the LSS gradient steepness, given by:

(5) $$b=S\times s^{*}$$

where s* is the normalized gradient slope, which can be expressed as:

(6) $$s^{*}=\frac{t_{0}\times \Delta C}{t_{g}}$$

Here, *t*_0_ is the column dead time (column dead volume to flow‐rate ratio, *V*_0_/*F*), ΔC is the difference in the volume fractions of the organic modifier between final and initial gradient conditions, and *t*_g_ is the gradient time. The s* term is therefore expressed without units. The column dead volume (*V*_0_) was estimated using uracil dissolved in ACN:H_2_O (80:20) and analyzed using the same mobile phase conditions (ACN:H_2_O, 80:20) to ensure that it cannot be retained under RPLC conditions. *V*_0_ was estimated by subtracting the extra‐column volume from the retention volume of uracil.

By assuming 1/*k*_i_ negligible (compound strongly retained under initial gradient conditions), Equation (4) can be simplified into:

(7) $$k_{e}=\frac{1}{2.3\times S\times s^{*}}$$

When combining Equations (3) and (7), the following equation can be obtained:

(8) $$C_{e}=\frac{1}{S}\log\left(s^{*}\right)+\frac{1}{S}\log\left(2.3\times S\right)+\frac{1}{S}\log\left(k_{0}\right)$$

As shown in Equation (8), the expression of *C*_e_ versus log s* is a linear expression *C*_e_ = αlog (s*) + β, with *S* and log *k*_0_ related to the slope (α) and the intercept (β) by the following relationships:

(9) $$S=\frac{1}{\alpha }$$

(10) $$\log\left(k_{0}\right)=S\times \beta -\log\left(2.3\times S\right)$$

It is important to keep in mind that *C*_e_ versus log s* is well fitted by a straight line (*R* ^2^ close to 1) provided that *k*_i_ values are sufficiently large (first hypothesis, see Equation (7)) and the retention model is linear (second hypothesis, see Equation (1)). In this sole case, accurate values of *S* and log *k*_0_ can be directly extracted from the slope (α) and the intercept (β), as shown by Equations (9) and (10).

**FIGURE 1:**
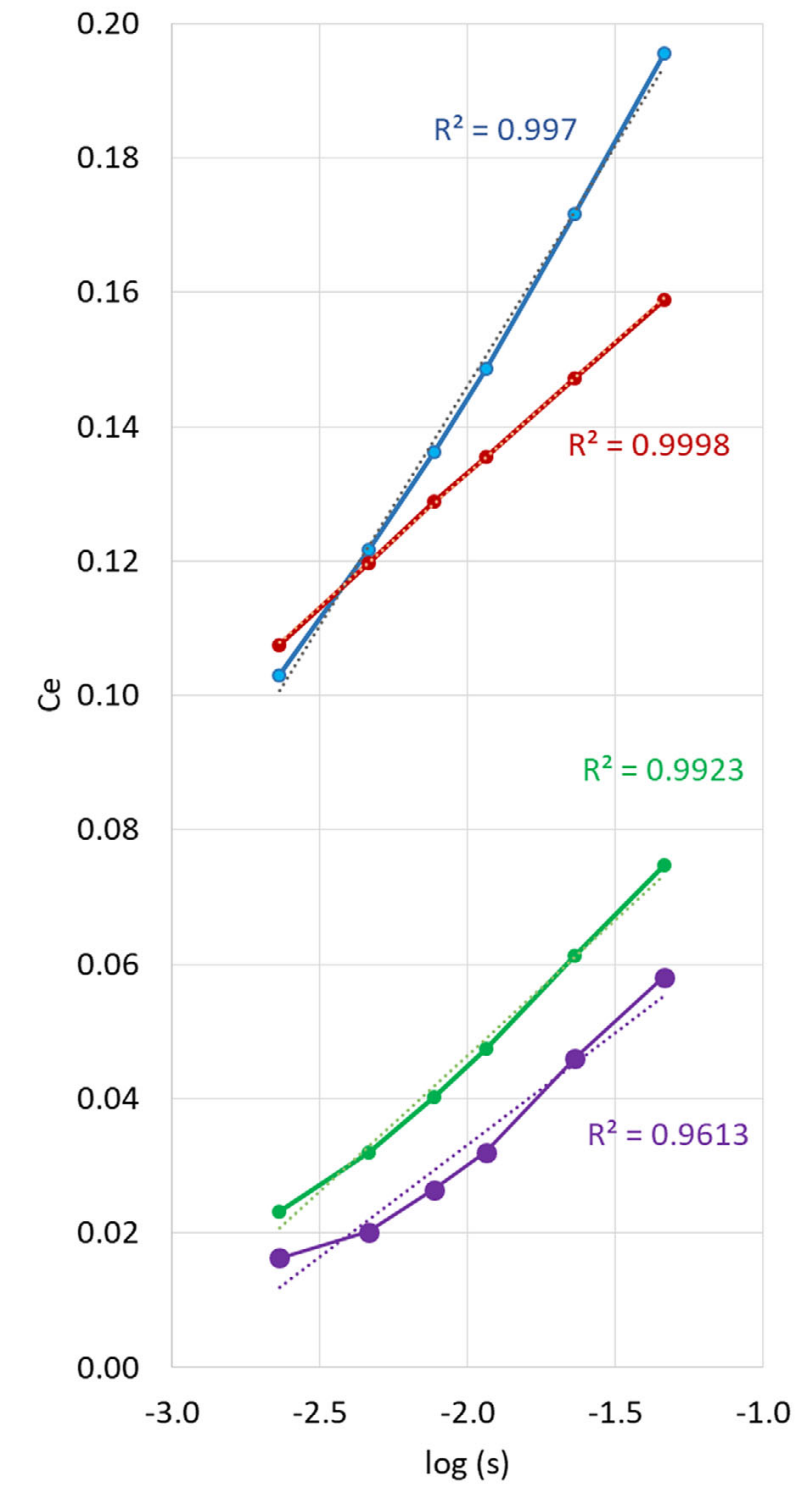
Representation of *C*_e_ versus log s*. This representation is used to calculate the linear solvent strength (LSS) parameters, with our new mathematical treatment based on the slope (α) and intercept (β) of the curves (Equation (8)). Four different peptides were considered as representative examples, offering small or large *k*_i_, and also some more or less linear retention models

The plots of experimental *C*_e_ versus log s* and the associated straight lines are shown in Figure 1 for four different peptides. As shown in Figure 1, the behavior of the four selected peptides is clearly different, with *R* ^2^ ranging from 0.96 (purple) to 0.9998 (red). The behavior of these peptides illustrates the four scenarios that can be encountered: (i) for the red peptide (*R* ^2^ close to 1), both hypotheses are verified (large k_i_ and linear retention model), which makes possible the accurate calculation of coefficients; (ii) with the blue peptide, only the first hypothesis is verified, but the model is not sufficiently linear; (iii) for the green peptide, the model is linear, but k_i_ is very low, and (iv) with the purple peptide, neither hypothesis is verified, leading to a critical situation. For the purple peptide, our method may be very difficult (impossible) to apply. However, it is not clear whether it can be successfully applied to the three other peptides. Therefore, this figure highlights the need to establish criteria to know the limit of the method.

### 2.2 Calculation of errors on predicted retention times

The accuracy of retention model prediction is generally assessed by the error on predicted *vs*. experimental retention times, which can be calculated using the following simple equation:

(11) $$\mathrm{Error}\;\left(\%\right)=\frac{t_{r,\mathrm{predicted}}-t_{r,\mathrm{experimental}}}{t_{r,\mathrm{experimental}}}\times 100$$

It is usually admitted that this error should not be higher than 2% [11] (this error is based on the routine industrial practice for retention time deviation of five injections of an SST solution, which should provide an RSD value < 2%) which means, under isocratic conditions, that the resolution may vary by 0.5 for a standard column producing 10 000 plates.

In gradient elution, the retention time error calculated with Equation (11) is meaningless in terms of resolution, since the peak widths do not vary according to the retention factor (*k*), but rather according to the *b* value, which depends on both *S* (often very similar values for similar compounds) and s*. Since all the experiments reported in this work were performed in gradient elution mode, we, therefore, estimate the error with another equation, which considers the ratio between the time difference and the calculated peak width in a gradient mode (*w*, calculated at 4σ), according to:

(12) $$\lambda =\frac{t_{r,\mathrm{predicted}}-t_{r,\mathrm{experimental}}}{w}$$

where

(13) $$w=\frac{4t_{0}}{\sqrt{N}}\times \left(\frac{1+2.3b}{2.3b}\right)$$

where *N* is the maximum plate number that can be reached with the selected column (van Deemter minimum).

In other words, λ corresponds to the chromatographic resolution achievable between the experimental and predicted peaks. Accordingly, this error is quite relevant for comparing different compounds (different *S* values) and/or different gradient conditions (different s* values).

Based on the definition of acceptable Error% (equal to 2%, as previously discussed), a maximum λ value of 0.5 was considered for accurate retention time predictions. Therefore, if the predicted resolution between two peaks is 1.5, the experimental resolution could be as low as 1, to be considered acceptable.

To better understand the differences between the error values obtained with Equation (11) (Error%) and Equation (12) (λ) in the case of gradient experiments, we have calculated these two different values for the set of 36 peptides obtained from the tryptic digestion of the six model proteins (see Materials and Methods section) and reported the corresponding values in Figure S1. In this figure, the λ values divided by 0.5 were located on the x‐axis, while the Error% values divided by 2 were reported on the y‐axis. With this representation, normalized/comparable values (without units) can be obtained on both axes, since a resolution of 0.5 between experimental and predicted retention times corresponds to an error value on retention time equal to 2%. The two normalized values can then be directly compared. Error% values reported in Figure S1 correspond to the predictions made for t_G5_ (longest gradient) with the HPLC modeling software based on t_G1_ and t_G4_ (intermediate gradient times, see Table S1 for more information on gradient times). From this figure, it is clear that the Error% values (y‐axis) were always minimized compared to λ (x‐axis), as all the Error% values were located below the reference line corresponding to equivalent results for Error% and λ (error of 2% *vs*. resolution of 0.5). As highlighted by the blue circle in Figure S1, the differences between the two error values calculated with Equations (11) and (12) were particularly important when the compounds were strongly retained (large t_r_) or when thin peaks were obtained (small S value).

The error λ was therefore considered in this study with a threshold value of 0.5, beyond which the predictions are uncertain.

## 3 MATERIALS AND METHODS

### 3.1 Chemicals and reagents

Type 1 water was provided by a Milli‐Q purification system from Millipore (Burlington, MA, USA). The mobile phase components, namely formic acid (FA), trifluoroacetic acid (TFA), methanol (MeOH) and ACN were obtained from Sigma Aldrich (Steinheim, Germany). The small molecules analyzed in this work: atenolol, caffeine, nadolol, propranolol, ibuprofen, methylparaben, ethylparaben, propylparaben, and butylparaben were also obtained from Sigma‐Aldrich. The six proteins digested to obtain the tryptic digest analyzed in this work, namely human serum albumin (HSA), BSA, β‐casein, myoglobin, lysozyme, and cytochrome C were all obtained from Sigma‐Aldrich. The intact proteins, namely insulin, α‐lactalbumin, and HSA were obtained from Sigma‐Aldrich, while rituximab was obtained as European Union pharmaceutical‐grade drug products from its respective manufacturer (Roche, Basel, Switzerland). Finally, some reagents such as DL‐1,4‐dithiothreitol and iodoacetamide were obtained from Acros Organics (Geel, Belgium), while trypsin was obtained from Sigma‐Aldrich.

### 3.2 Sample preparation

The 36 model peptides for which LSS parameters were calculated were obtained by tryptic digestion of six proteins (HSA, BSA, β‐casein, myoglobin, lysozyme, and cytochrome C) following a protocol described elsewhere [12, 13]. The various peptides were selected to have as diverse *m/z* ratios as possible, and the peptides providing the most intense MS response were preferentially selected.

The light chains (LC, 25 kDa) and heavy chains (HC, 50 kDa) of rituximab were obtained after the reduction of the interchain disulfide bridges by the addition of 100 mM of DL‐1,4‐dithiothreitol solution to 1 mg/ml of protein material and incubating for 30 min at 45°C [14].

### 3.3 Instrumentation and chromatographic conditions for the analysis of small molecules and peptides

The experiments relative to peptides and small molecules were performed on a UHPLC 1290 Infinity system from Agilent Technologies (Waldbronn, Germany). The binary system was equipped with two high‐pressure solvent delivery pumps, an autosampler with a flow‐through needle injector, and a thermostatic column compartment with low dispersion preheaters. The system was hyphenated to a quadrupole‐TOF high‐resolution mass spectrometer (model G6560B) from Agilent Technologies. The measured dwell volume and overall extra‐column volumes were 0.17 and 0.031 ml, respectively. Instrument control and data acquisition were performed by Mass Hunter software (Agilent Technologies). Mass spectrometry data were acquired in positive ion mode for peptides and basic compounds, and negative mode for acidic compounds. The acquisition rate was 20 spectra/s.

The chromatographic column was an Acquity CSH C18 (50 × 2.1 mm, 1.7 μm) from Waters (Milford, MA, USA). Mobile phase A was composed of H_2_O + 0.1% FA, while mobile phase B contains ACN + 0.1% FA. An equilibration time of five dead times was systematically added between runs. The column temperature was 80°C for peptides with a flow rate of 2.1 ml/min. It was 30°C for small molecules with a flow rate of 0.5 ml/min.

### 3.4 Instrumentation and chromatographic conditions for the analysis of proteins

The experiments relative to proteins and monoclonal antibodies were performed on an Acquity UPLC system (Waters). The system was equipped with a binary solvent delivery pump, an autosampler, a UV detector with a 0.5 μl flow cell (wavelength of 214 nm, 20 Hz data acquisition rate, and fast time constant), and an injector equipped with a 2 μl injection loop (weak solvent was a mixture of H_2_O:ACN 90:10, strong solvent was pure ACN). The overall extra‐column volume was about 13 μl as measured from the injection seat of the auto‐sampler to the detector cell. The measured dwell volume was 0.1 ml. Data acquisition and instrument control were performed by Empower Pro 2 software (Waters).

The chromatographic column was a Waters bioresolve RP mAb polyphenyl (100 × 2.1 mm, 2.7 μm, 450 Å). Mobile phase A was composed of H_2_O + 0.1% TFA, while mobile phase B contains ACN + 0.1% TFA. An equilibration time of 10 min was systematically added between runs. The flow rate was equal to 0.5 ml/min, while the mobile phase temperature was equal to 80°C.

### 3.5 Experimental procedure

Initial and final gradient compositions (*C*_i_ and *C*_f_) were determined from preliminary runs for the different types of compounds (i.e., small molecules, peptides, and proteins). The goal was to obtain a suitable retention window, whatever the gradient time.

Six different gradient times (*t*_G0_, *t*_G1_, *t*_G2_, *t*_G3_, *t*_G4_, and *t*_G5_) were considered for small molecules using either ACN or MeOH and for peptides with ACN. The different gradient times corresponded to *b* values (Equation (5)) ranging from about 0.05 (slowest gradient) to 1 (fastest gradient). For proteins, only five gradient times (*t*_G1_, *t*_G2_, *t*_G3_, *t*_G4_, and *t*_G5_) were considered, and b values ranged from 0.05 to 0.5 (*b* value of 1 was unrealistically fast and therefore discarded). All the corresponding initial and final compositions as well as gradient times for the different samples are reported in Table S1 (supplementary material).

The composition at elution, *C*_e_ was determined from the experimental retention time (*t*_r_) according to the following equation:

(14) $$C_{e}=C_{i}+\frac{\left(C_{f}-C_{i}\right)}{t_{G}}\left(t_{r}-t_{0}-t_{D}\right)$$

where *C*_i_ and *C*_f_ are the initial and final compositions respectively and *t*_D_, the instrument dwell time. It is important to mention that the dwell time was estimated using the approach involving the use of water in the A‐solvent reservoir and water + 0.1% acetone in the B‐reservoir, after replacement of the column with a tubing generating about 50 bar, to have the pump working properly.

The predicted retention time (*t*_r_), was determined from calculated *S* and log *k*_0_ according to:

(15) $$t_{r}\;=\;\frac{t_{0}}{b}\log(2.303\times k_{i}\times b\times \left(1-t_{D}/\left(t_{0}\times k_{i}\right)+1\right)+t_{0}+t_{D}$$

**FIGURE 2:**
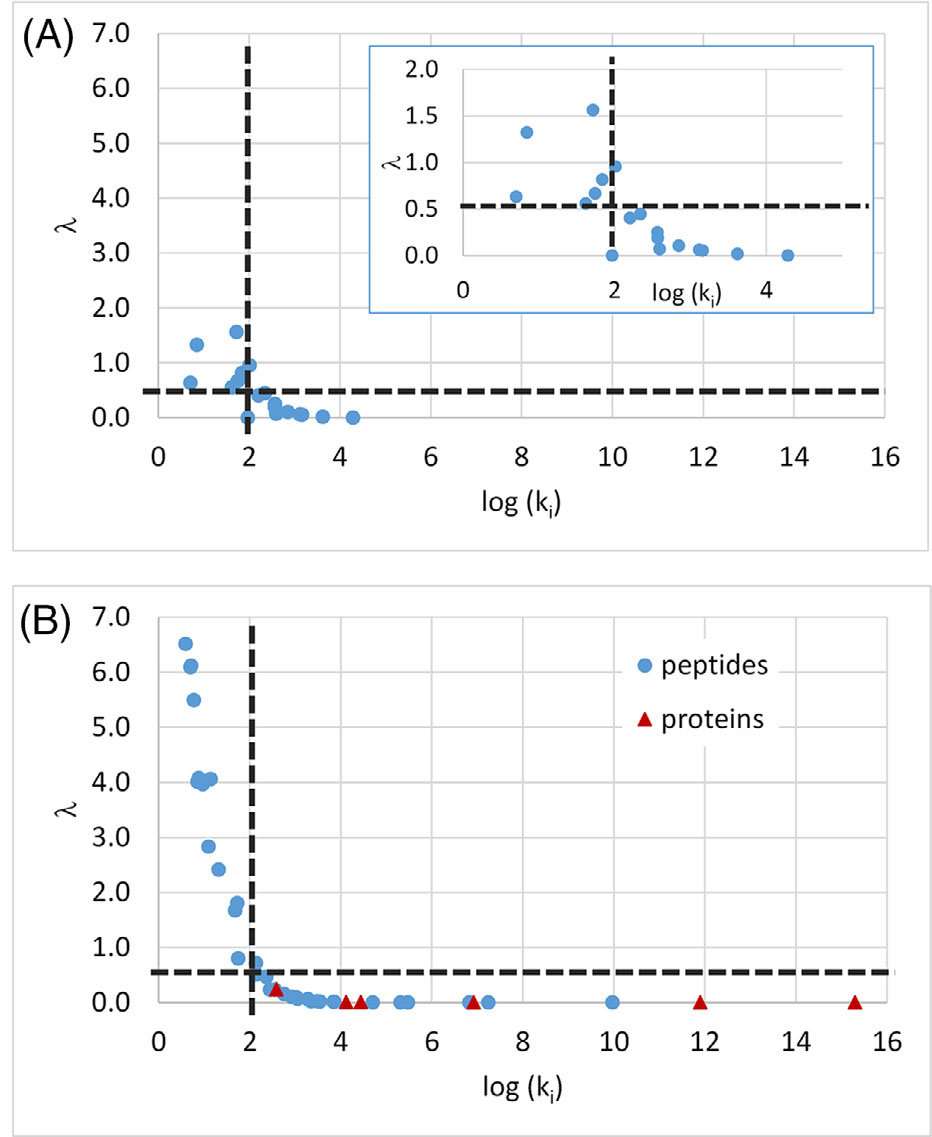
Accuracy of the predicted retention times obtained with the new mathematical treatment as a function of log *k*_i_. The error values (*λ*) were calculated for (A) nine small molecules using ACN or methanol (MeOH) as an organic modifier, and (B) 36 peptides and six proteins

**FIGURE 3:**
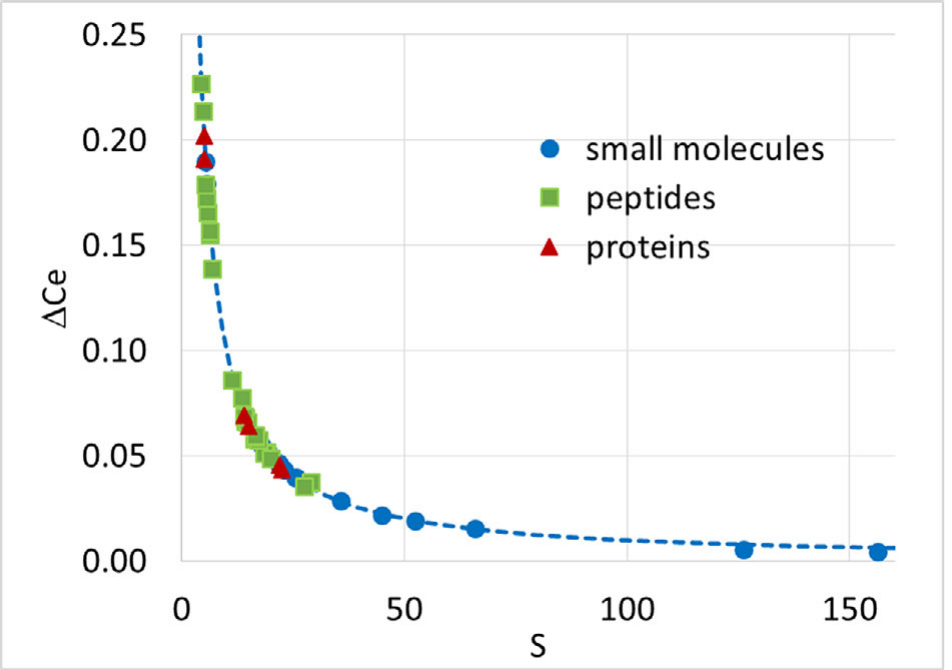
Variation of elution composition ΔC_e_ between two gradient times (*t*_G1_ and *t*_G5_) as a function of *S* value (obtained by calculation from *t*_G1_ and *t*_G4_). Various types of molecules were considered (small molecules, peptides, and proteins)

**FIGURE 4:**
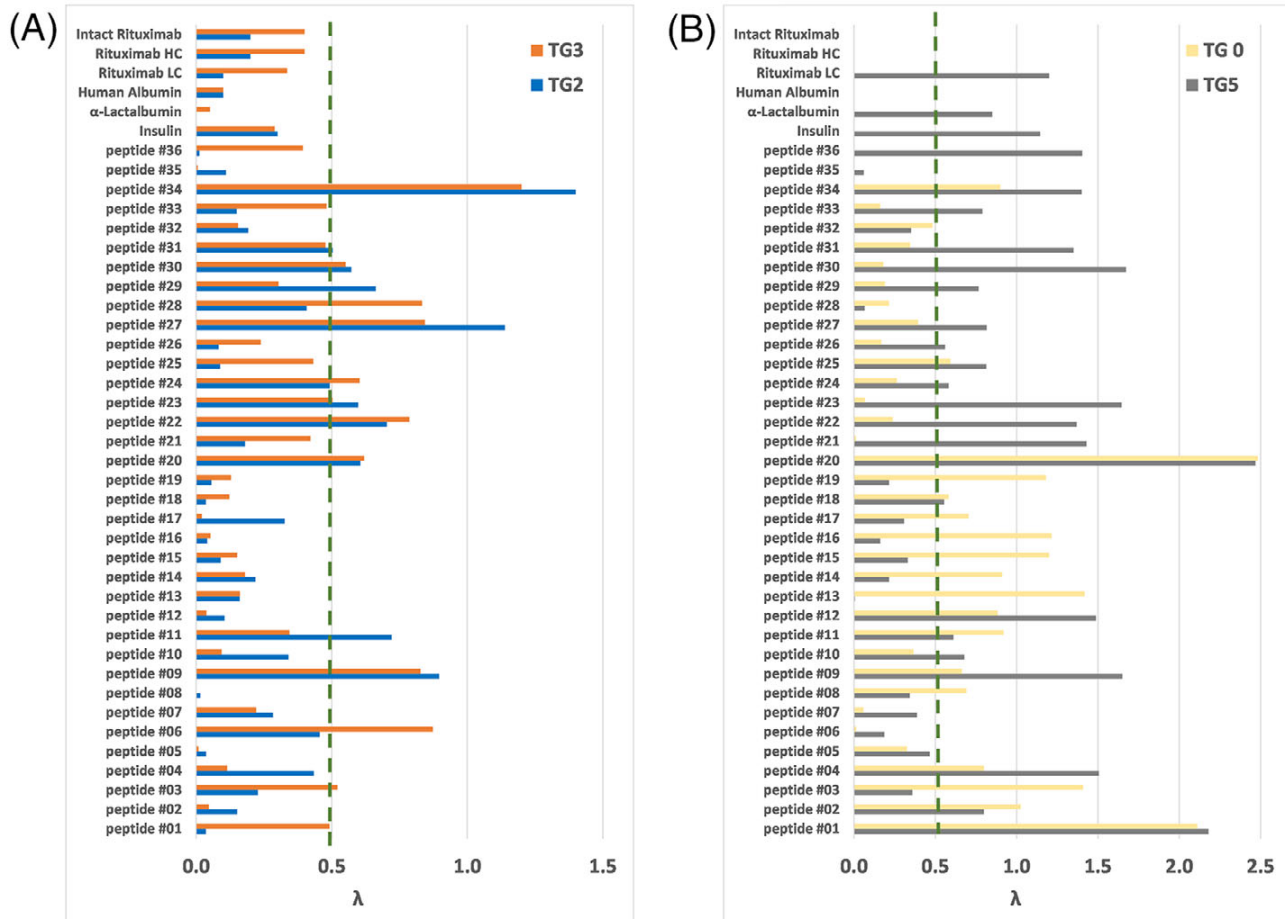
Evaluation of retention models linearity for 36 peptides and six proteins. (A) Predicted retention times were calculated by interpolation for two different gradient times, *t*_G2_ and *t*_G3_, based on *t*_G1_ and *t*_G4_, (B) predicted retention times were calculated by extrapolation for two different gradient times, *t*_G0_ and *t*_G5_, based on *t*_G1_ and *t*_G4_

**FIGURE 5:**
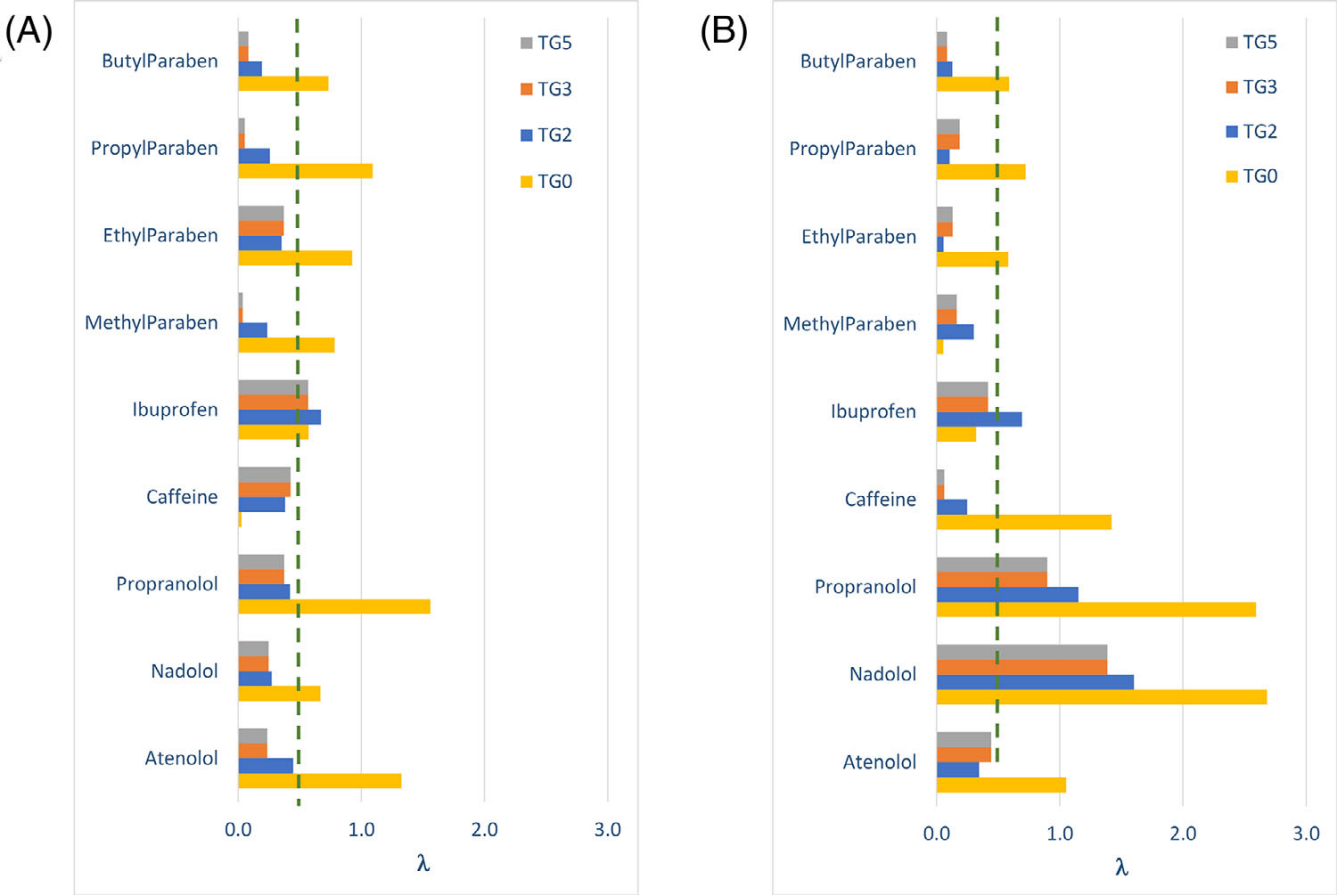
Evaluation of retention models linearity for nine small molecules. Predicted retention times were calculated for four different gradient times, *t*_G0_, *t*_G2_, *t*_G3_, and *t*_G5_, based on *t*_G1_ and *t*_G4_ (interpolation and extrapolation). Two different organic modifiers were considered in the mobile phase: (A) methanol (MeOH) and (B) ACN

All the reported values in Figures 2, 3, 4, 5 were calculated from *t*_G1_ and *t*_G4_, either using the new mathematical treatment explained in Section 2.1 or with the commercial HPLC modeling software, Osiris (Euradif, France).

## 4 RESULTS AND DISCUSSION

### 4.1 Applicability of the new method

In the first instance, the ability of the new mathematical treatment to accurately calculate log *k*_0_ and *S* values was established for various compounds belonging to different classes (i.e., small molecules, peptides, and proteins). Two gradient runs (*t*_G1_ and *t*_G4_) and the corresponding experimental retention times were used for the calculation, as detailed in Section 2.1. The retention times of all compounds, predicted for *t*_G4_ with Equation (15) were compared to the experimental ones. The corresponding λ error between predicted and experimental retention times can only come from a wrong assumption on large values of *k*_i_, but not from a possible non‐linear model since any compound with sufficiently large *k*_i_ should still have a zero error with this gradient time (as duly checked with Osiris software). As described in Section 2.1, our mathematical treatment is only valid as long as log *k*_i_ is sufficiently high (Equation (7)). To assess the limits of the method, we have plotted the λ values versus log *k*_i_ for small molecules (Figure 2A) and for peptides/proteins (Figure 2B). As previously discussed, a cut‐off value of 0.5 for λ, was considered to check whether the prediction error is too large. A value of λ above 0.5 means that the new calculation method cannot be successfully applied.

In the case of small molecules (Figure 2A), all the λ values were below 1.6, whatever the analyte. The data points were scattered and a general trend cannot be easily found. However, it is also important to notice that all the λ values were below 0.5 when log k_i_ was higher than 2.1, which corresponds to a *k*_i_ value of about 125. It is therefore recommended to perform the preliminary gradients (used to calculate the linear model coefficients) starting with the lowest possible initial composition, to have the best chance of meeting the requirements in terms of high *k*_i_ values.

In the case of biomolecules (peptides and proteins), the representation of λ versus log *k*_i_ is provided in Figure 2B using a different color code for peptides and proteins. As can be observed, λ values can be as high as 6.5, which is a much higher value than for small molecules (λ < 1.5). In addition, a strong relationship was observed between λ and log *k*_i_. Based on these observations, it is clear that the initial solvent strength for preliminary gradients must be low enough since λ error is more critical than for small molecules, otherwise the coefficients calculated with the method described in Section 2.1 can be highly inaccurate. Here again, the same conclusion can be drawn in terms of the log k_i_ cut‐off value (i.e., > 2.1) allowing to obtain a reasonable error (λ < 0.5). In the case of large molecules, such as proteins, k_i_ values are expected to be very large (see Figure 2B, where log k_i_ can be as high as 15 in some cases), due to the on/off retention mechanism of proteins [15]. Indeed, it has been shown that the transition range between a fully adsorbed and desorbed state of proteins at the surface of the column corresponds to a %ΔC of only 3.5% for an intact mAb of 150 kDa [16]. This special chromatographic behavior of proteins explains why the methodology described in Section 2.1 is more readily applicable to proteins and riskier with small molecules. For example, with intact rituximab, light and heavy chains of rituximab, human albumin, and α‐lactalbumin (all species have MW > 14 kDa), λ values were all very close to 0, which means that the estimation of *S* and log *k*_0_ does not bring any error. With the smallest protein (insulin, MW = 5808 g/mol), λ was slightly higher (λ = 0.2), but still fully acceptable.

According to Equation (8), the difference in composition at elution (ΔC_e_) for a given compound between two different normalized gradient slopes is expressed by:

(16) $$\Delta C_{e}=\frac{1}{S}\log\left(\frac{s_{2}^{*}}{s_{1}^{*}}\right)$$

Figure 3 shows the plot of ΔC_e_ against the calculated *S* values for our studied compounds. The analytes with log *k*_i_ < 2.1 were not considered, since the calculated *S* values are expected to be incorrect. In this case, the ratio of normalized gradient slopes was 10, thus corresponding to a ratio of 10 between the gradient times (i.e., *t*_G1_ and *t*_G5_). The considered molecules include the six proteins, the 36 peptides, and the nine small molecules eluted with both MeOH and ACN. The theoretical curve obtained from Equation (16) (which depends only on the selected s* ratio) is shown in a dotted line.

In this figure, it is interesting to compare ΔC_e_ and *S* values for the different types of analytes. For the proteins (red triangles), ΔC_e_ was on average equal to 1.7%, while this value increases to 5.2% for peptides (green square) and 15.3% for small molecules (blue circle). Interestingly, for the two largest proteins, namely intact mAb, and human albumin, ΔC_e_ was equal to 0.57 and 0.45%, respectively. This means that the percentage of ACN at the elution time remains almost identical whatever the gradient conditions applied. In isocratic elution, a very small change in the percentage of ACN would make these proteins go from zero retention to almost infinite retention (on‐off retention mechanism). Obviously, this is not true with small molecules, for which a ΔC_e_ of more than 20% was observed (see the case of parabens) when using MeOH as an organic modifier.

Finally, it is important to note that the rule of thumb (log *k*_i_ > 2.1) makes it possible to sort out the molecules for which our mathematical treatment is valid. For example, in Figure 1, log k_i_ values were above 2.1 for both red and blue peptides (3.6 and 2.4, respectively), while they were below 2.1 for both green and purple peptides (1.4 and 0.85, respectively). However, as stated before, at this stage, we do not know whether the retention models are linear or not.

### 4.2 Assessment of model linearity and implication on coefficient calculations

The next step to check the applicability of our mathematical treatment is to consider a possible non‐linearity of the retention models. Indeed, whatever the number of initial gradients considered for retention modeling, the calculation of log *k*_0_ and *S* values described in Section 2.1 involves a linear regression, and Equation (8) is only valid as long as the retention model is linear. Thus, if the retention model is quadratic or even more complex, it is possible that the retention times prediction may be inaccurate.

The first assumption (large k_i_) is not required by Osiris software to accurately calculate the two coefficients of the linear model (Equation (1)) from two gradient runs. We, therefore, compared the retention times, predicted by Osiris, to the experimental ones, to assess the model linearity. Unlike with our new methodology, a low *k*_i_ value had no impact on the retention time prediction.

Two preliminary gradients (*t*_G1_ and *t*_G4_) were considered for the calculation with small molecules, peptides, and proteins. The retention times were predicted for *t*_G2_ and *t*_G3_ (interpolation inside the *t*_G1_ − *t*_G4_ range) as well as for *t*_G0_ and *t*_G5_ (extrapolation outside the *t*_G1_ − *t*_G4_ range).

Figure 4 shows the corresponding results for peptides and proteins. As expected, the λ error values were very good by interpolation (Figure 4A), despite a ratio of 5 between the two gradient times (*t*_G1_ corresponds to a *b* value of 0.5, while *t*_G4_ corresponds to 0.1). It should be noted that the usual ratio recommended for accurate retention modeling is 3 only [10, 17], thereby leading to a smaller interpolation range. In terms of retention time prediction for *t*_G3_, more than 90% of the λ values were below 0.5, while only one peptide has a λ value higher than 1. Again, Figure 4A confirms that our new calculation procedure was particularly well suited for proteins since all λ values were comprised between 0.1 and 0.4. This suggests that the retention models of proteins are more linear than those of peptides. The prediction results were less good by extrapolation (Figure 4B), confirming that retention models can be considered linear only within a limited retention window. This is particularly true for the two peptides (#1 and #20) offering λ values higher than 2, such value corresponding to the bad case where predicted and experimental peaks would be fully baseline resolved. The vast majority of peptides exhibit λ values above 0.5, either for *t*_G0_ or for t_G5_. Even in the case of proteins, the errors can reach λ value up to 1 by extrapolation. However, it is important to mention that the λ values calculated with Equation (12) can be overestimated in the case of proteins since the calculation is based on the average peak width value (*w*), which considers the maximum plate number, (*N*, van Deemter minimum) that can be obtained with the selected column. Obviously, with proteins, *N* values are expected to be much lower at the (non‐optimal) flow rate that was used in this work, leading to higher w values, and therefore lower λ values. Interestingly, there are numerous examples where an extrapolation to t_G5_ provides an acceptable error, but the extrapolation for the same molecule to *t*_G0_ offers a λ error much higher than 0.5. The reverse scenario (good extrapolation with *t*_G0_ and worse results with *t*_G5_) was also widely observed. This confirms that extrapolation should be avoided or at least critically used, as it is a risky procedure.

Figure 5 shows the retention time predictions for small molecules (i.e., neutral, acidic, and basic) when using MeOH (Figure 5A) or ACN (Figure 5B) as organic modifiers in the mobile phase. When interpolation was considered (prediction for *t*_G2_ and *t*_G3_), it appears that the models were sufficiently linear for accurate retention time prediction with MeOH, while the λ values were sometimes higher than 0.5 with ACN, in particular for basic compounds (β‐blockers). The fact that the linear model was more accurate with MeOH versus ACN has already been previously described in the literature [10]. When considering extrapolation (prediction for *t*_G0_ and *t*_G5_), the predicted retention times were always less accurate (similarly to what was observed in Figure 4B), and about 50% of the λ values were higher than 0.5 with MeOH, while this value increases to more than 80% with ACN as the organic modifier. The situation was particularly critical with basic compounds, for which λ values were always higher than 2.

Based on the observations made in Figures 4 and 5, it is clear that the non‐linearity is more critical for accurate predictions by interpolation for small molecules and peptides, compared to proteins. However, it also appears that extrapolation should not be considered, whatever the type of compound, as the risk to obtain inaccurate predictions of retention times becomes too high.

## 5 CONCLUSIONS

This works highlights the development of a simple mathematical treatment based on linear regression for the rapid determination of LSS parameters in RPLC from a minimum of two initial gradients. This strategy is a useful alternative to the use of HPLC modeling software, for those who are not equipped. To verify the applicability of the methodology, various molecules (i.e., small compounds, peptides, and proteins) were considered, and various gradient conditions were realized. The retention time prediction accuracy, based on LSS behavior was evaluated from the calculation of the λ value, which corresponds to the chromatographic resolution between the experimental and predicted peaks in gradient mode. If the λ value is below 0.5, the retention time prediction was considered sufficiently accurate.

In most of the cases, highly accurate predictions of log *k*_0_ and *S* values were obtained (similar to what can be obtained with HPLC modeling software), but two important constraints have to be considered. First, the log *k*_i_ values (retention factor under initial gradient conditions) have to be at least equal to 2.1, to keep the error on retention time prediction reasonable (Equation (7) is only valid when this condition is met), which means that the initial gradient composition of the preliminary gradients has to be sufficiently low. Due to the on‐off retention mechanism observed for proteins, this condition was easily reached for large biomolecules, while it was not always the case with small molecules. Secondly, the retention models have also to be as linear as possible for an accurate prediction of retention times, otherwise, Equation (8) is not valid. Here again, we proved that the models were always more linear for proteins versus peptides or small molecules. However, for an accurate retention time prediction, interpolation has to be preferred over extrapolation, and therefore the two gradients allowing the determination of the linear model coefficients have to be carefully selected.

Based on these observations, it is clear that the methodology presented in this manuscript is particularly interesting to further improve the retention prediction of biomolecules such as proteins, for which the values of *S* and *k*_0_ are so large that it is sometimes difficult to obtain sufficient precision with some commercial HPLC modeling software. Moreover, with this mathematical treatment, the calculations of LSS parameters can also be performed based on three or more initial gradients without any additional difficulty (simple fitting of a linear model). When increasing the number of initial gradients, the fitting will be more precise and so does the values of log *k*_0_ and *S*.

Finally, it is important to notice that accurate log *k*_0_ and *S* of proteins can be particularly relevant in the following cases: i) method optimization to improve selectivity between critical peak pairs [18, 19], ii) development of multi‐isocratic experiments [20, 21], and iii) finding the best combination of columns for serial coupling [22].

A simple Excel tool was developed for rapid and accurate calculation of all LSS parameters (the tool is particularly adapted for proteins, as shown in this work), and is freely available on our website [23]. This Excel spreadsheet is also available as supplementary material for this article.

## CONFLICT OF INTEREST

The authors have declared that there is no conflict of interest.

## Supporting information



Supporting Informtion 1

Supporting Informtion 2

## Data Availability

The data that support the findings of this study are available in the supplementary material of this article.
