# Supplementary material for: A simple mathematical treatment for predicting linear solvent strength behavior in gradient elution: Application to biomolecules
Source: J Sep Sci. 2022 May 26;45(17):3276–85. doi: 10.1002/jssc.202200161 (PMC9543774; doi:10.1002/jssc.202200161)
Supplement: Supplementary file 1 — Supporting Informtion 1 [file JSSC-45-3276-s002.docx]

**A simple mathematical treatment for predicting linear solvent strength behaviour in gradient elution: application to biomolecules**

Davy GUILLARME^1,2^, Thomas BOUVAREL^1,2^, Florent RIVIERE^3^, Sabine HEINISCH^3^

^1^ School of Pharmaceutical Sciences, University of Geneva, CMU - Rue Michel Servet 1, 1211 Geneva 4, Switzerland

^2^ Institute of Pharmaceutical Sciences of Western Switzerland, University of Geneva, CMU - Rue Michel Servet 1, 1211 Geneva 4, Switzerland

^3^ Université de Lyon, Institut des Sciences Analytiques, UMR 5280, CNRS, 5 rue de la Doua, 69100 Villeurbanne, France

**Supplementary material**

**Figure S1**: Comparison of two methodologies for the calculation of errors on predicted retention times under gradient mode. Based on Equations 11 and 12, the two values Error% and λ were normalized. The values of (Error%/2) were plotted as a function of (λ/0.5) for 36 different peptides. The blue circle highlights some significant differences between the two calculation methods.

**Table S1:** Gradient conditions (t_G0_, t_G1_, t_G2_, t_G3_, t_G4_, and t_G5_) used to analyze all the different compounds (small molecules, peptides, and proteins) presented in this work.

|  | **Approximate b value** | | **1** | **0.5** | **0.3** | **0.15** | **0.1** | **0.05** |
| --- | --- | --- | --- | --- | --- | --- | --- | --- |
|  | **Gradient time (min)** | | **tG0** | **tG1** | **tG2** | **tG3** | **tG4** | **tG5** |
|  | **%initial** | **%final** |  |  |  |  |  |  |
| **Ibuprofen** | 1 | 99 | 1.5 | 3 | 5 | 10 | 15 | 30 |
| **Methylparaben** | 1 | 99 | 1.5 | 3 | 5 | 10 | 15 | 30 |
| **Ethylparaben** | 1 | 99 | 1.5 | 3 | 5 | 10 | 15 | 30 |
| **Porpylparaben** | 1 | 99 | 1.5 | 3 | 5 | 10 | 15 | 30 |
| **Butylparaben** | 1 | 99 | 1.5 | 3 | 5 | 10 | 15 | 30 |
| **Atenolol** | 1 | 70 | 1.5 | 3 | 5 | 10 | 15 | 30 |
| **Nadolol** | 1 | 70 | 1.5 | 3 | 5 | 10 | 15 | 30 |
| **Propranolol** | 1 | 70 | 1.5 | 3 | 5 | 10 | 15 | 30 |
| **Caffeine** | 1 | 70 | 1.5 | 3 | 5 | 10 | 15 | 30 |
| **Tryptic digest** | 1 | 41 | 0.5 | 1 | 2 | 3 | 5 | 10 |
| **Insulin** | 20 | 40 | - | 5.0 | 8.4 | 16.8 | 25.2 | 50.5 |
| **α-Lactalbumin** | 25 | 45 | - | 7.1 | 11.8 | 23.5 | 35.3 | 70.6 |
| **Human albumin** | 25 | 45 | - | 22.3 | 37.1 | 74.3 | 111.4 | 222.8 |
| **Rituximab LC** | 25 | 45 | - | 8.4 | 14.0 | 28.0 | 42.0 | 83.9 |
| **Rituximab HC** | 25 | 45 | - | 9.0 | 15.0 | 30.1 | 45.1 | 90.3 |
| **Intact rituximab** | 25 | 45 | - | 25.0 | 41.7 | 83.4 | 125.0 | 250.1 |
